# In Memoriam: Joshua Lederberg (1925–2008)^1^

**DOI:** 10.3201/eid1406.080413

**Published:** 2008-06

**Authors:** James M. Hughes, D. Peter Drotman

**Affiliations:** * Emory University, Atlanta, Georgia, USA; † Centers for Disease Control and Prevention, Atlanta

**Keywords:** Memoriam, Nobel laureate, emerging infectious diseases, Joshua Lederberg, commentary

**Figure:**
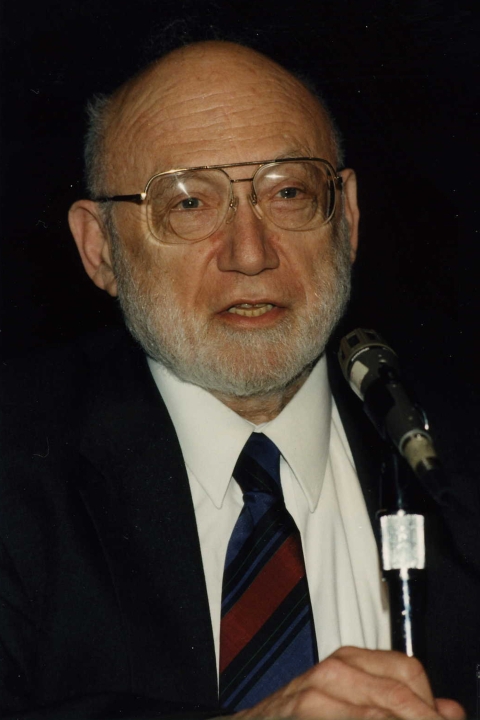
Dr Lederberg

The world of emerging infections lost a valuable friend and inspiring leader earlier this year with the death of Joshua Lederberg. The globally recognized scientist, educator, national and Presidential scientific advisor, and Nobel Laureate died of pneumonia on February 2, 2008, at the age of 82. Dr Lederberg’s early work on bacterial genetics virtually established the discipline of molecular biology, earning him a Nobel Prize in Physiology or Medicine in 1958, when he was only 33 years of age. His contributions paved the way toward understanding microbial adaptation, including the development of antimicrobial drug resistance and the implications of these evolutionary changes for clinical medicine and public health. Equally impressive are his public service contributions; he served as a science advisor to 9 US presidents.

Dr Lederberg began his exploration of bacterial genetics as a doctoral student at Yale University, where he forged new paths and gained prominence in the developing field. Over the next 30 years, he held academic posts and chaired new genetics departments at the University of Wisconsin and Stanford University. In 1978, he joined Rockefeller University as its president. Over the next 12 years, his accomplishments and successes in this position were a testimony to his scholarly accomplishments and leadership skills; he remained affiliated with the university for the rest of his life. He offered science policy advice not only to 9 US administrations but also to the National Aeronautics and Space Administration and the Department of Defense. In addition to the Nobel Prize, his numerous prestigious awards included the National Medal of Science in 1989 and the Presidential Medal of Freedom in 2006.

**Figure 1:**
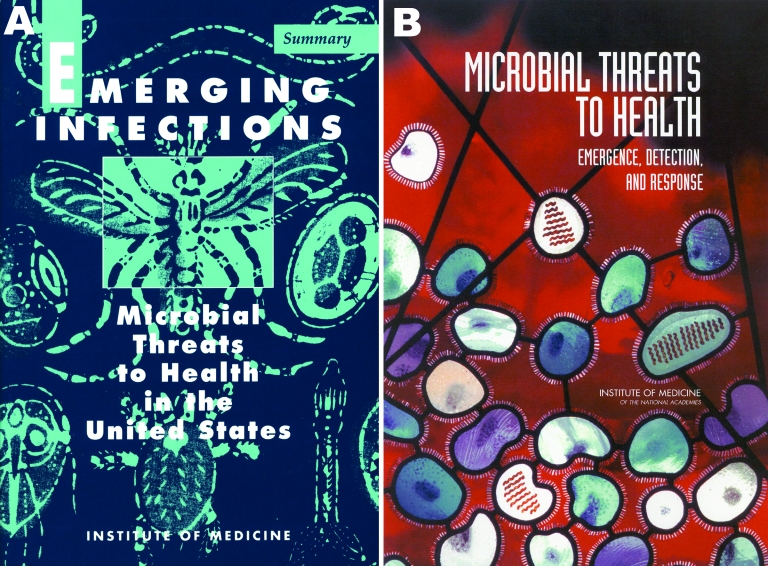
A) Emerging Infections: Microbial Threats to Health in the United States (*4*), a report of the Institute of Medicine (IOM) Committee on Emerging Microbial Threats to Health, published in 1992. B) Microbial Threats to Health: Emergence, Detection, and Response (*8*), a report of the IOM Committee on Emerging Microbial Threats to Health, published in 2003.

To those of us working in the field of emerging infectious diseases, Dr Lederberg will be most remembered for his critical role in recognizing the threats posed by emerging and reemerging infections and their implications for public health and national security. He is well known to readers of Emerging Infectious Diseases (EID) as a contributor to the journal (*1*,*2*) and for his leadership as co-chair with the late Robert Shope (*3*) of the Institute of Medicine (IOM) Committee on Emerging Microbial Threats to Health, which produced the 1992 report Emerging Infections: Microbial Threats to Health in the United States (*4*) (Figure 1, **panel A**). This landmark report defined the concept of emerging and reemerging infections, identified factors contributing to disease emergence and reemergence, and emphasized current and future challenges posed by infectious diseases. The report also highlighted deficiencies in our nation’s public health infrastructure and made recommendations on the need to strengthen surveillance systems, address new areas of research, provide multidisciplinary training for the next generation of scientists and public health workers, and establish new and enhance existing disease prevention and control programs. EID owes its genesis to this report.

Drs Lederberg and Shope were extremely effective in communicating the committee’s observations and recommendations to the scientific, public health, and public policy communities, generating broad and renewed interest in infectious diseases. The report had a profound impact on the Centers for Disease Control and Prevention (CDC), the National Institute of Allergy and Infectious Diseases, the Department of Defense, and other federal agency programs involved in addressing emerging infectious disease threats. The report’s emphasis on the need for interdisciplinary strategies and coordinated approaches led to the establishment of the Working Group on Emerging and Re-emerging Infectious Diseases under the auspices of the National Science and Technology Council Committee on International Science, Engineering and Technology. This Working Group was chaired by CDC Director David Satcher, and its deliberations led to vastly improved communication and collaboration among many federal agencies (*5*).

**Figure 2:**
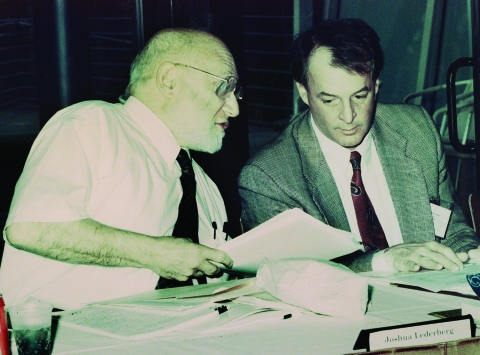
Institute of Medicine co-chair Joshua Lederberg (left) in conversation with James M. Hughes, director, National Center for Infectious Diseases, Centers for Disease Control and Prevention (CDC), during a meeting in 1993 with expert consultants on development of the first CDC emerging infectious disease strategy.

For CDC, the impact of the IOM report and Dr Lederberg’s contributions were substantial. CDC worked with partners to develop new domestic and global strategies to address emerging infections, including specific efforts to respond to the IOM recommendations. Dr Lederberg served as an advisor on the development of these strategies (Figure 2), the first of which was published in 1994 (*6*), with an update 4 years later (*7*). From 1994 through 2004, CDC’s funding for infectious diseases grew nearly 200-fold, from $1 million to >$190 million, enabling development and implementation of numerous programs to build epidemiology and laboratory capacity and improve preparedness and response capacity for infectious diseases and other health threats. Examples of these programs include the Emerging Infections Programs with activities such as FoodNet and Active Bacterial Core surveillance; the International Emerging Infections Programs; the Epidemiology and Laboratory Capacity for Infectious Diseases cooperative agreement; the Emerging Infectious Diseases Laboratory Fellowship Program; this journal; and the International Conference on Emerging Infectious Diseases, first held in 1998 with Dr Lederberg serving as the inaugural plenary speaker (*2*) and, most recently, in March 2008.

As an advocate for improvements in information systems and transparency, Dr Lederberg enthusiastically supported innovative approaches that included the establishment of ProMED and the Global Public Health Information Network. Internationally, he served as an advisor to the World Health Organization (WHO) on the creation of its Emerging Infections Program and personally advocated the need for establishing this program with WHO’s then director general, Dr Hiroshi Nakajima.

Dr Lederberg also served as co-chair, with Dr Margaret Hamburg, of a second IOM committee that produced a follow-up to the 1992 report. Microbial Threats to Health: Emergence, Detection, and Response was published in March 2003 (*8*), less than 1 week after WHO issued its first global health alert in response to the outbreak of severe acute respiratory syndrome (SARS). This report had a broader global perspective than the 1992 report and identified additional factors contributing to disease emergence, reemergence, and global spread. The report also anticipated many issues that the 2003 SARS outbreak so starkly illustrated, including the need for increased collaboration among the human and animal health communities, a recommendation now supported by the One Health Initiative (*9*). The 2003 report’s cover (Figure 1, **panel B**) depicts an artistic rendering of influenza virus, reflecting Dr Lederberg’s longstanding interest in influenza research and his concern about national and global preparedness for the next pandemic.

As a charter member of the IOM, Dr Lederberg was the driving force behind the creation of its Forum on Emerging Infections (now the Forum on Microbial Threats), steering it effectively as chairperson through 2002. Consistent with his approach, the forum comprises representatives of the medical, academic, public health, veterinary, agricultural, environmental, national security, and pharmaceutical sectors. An important part of his legacy, the forum continues to address a broad range of issues in an interdisciplinary setting on a regular basis, recently focusing on vector-borne and zoonotic diseases and the potential impact of climate change on infectious diseases. As Josh liked to say, there is an ongoing confrontation with the microbial world involving “our wits versus their genes.” The forum held a workshop in May 2008 on microbial evolution and coadaptation in honor of Dr Lederberg (www.iom.edu/CMS/3783/3924/52347.aspx).

On a personal level, I (J.M.H.) first encountered Professor Lederberg in the late 1960s, when I was a medical student and he was the chairman of the Genetics Department at Stanford. His stature and accomplishments were legendary, and we were in awe of him. Over the course of my CDC career, I was fortunate to have many interactions with Josh and to greatly benefit from his insightful questions, keen observations, and constructive comments. He was very approachable and consistently available to discuss a broad range of issues, having an uncanny ability to readily span from basic science, to applied science, to public health, to policy, and to national security. He was a mentor, friend, and colleague to many.

**Figure 3:**
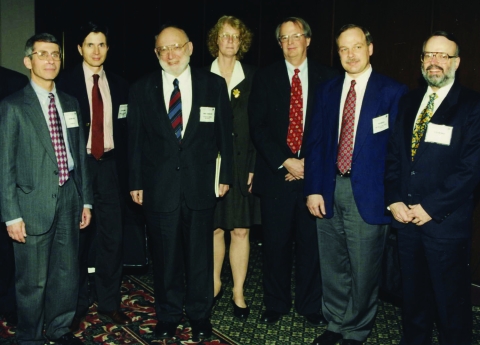
Keynote speakers at the inaugural International Conference on Emerging Infectious Diseases in Atlanta, March 8–11, 1998. Left to right: Anthony Fauci, David Heymann, Joshua Lederberg, Claire Broome, James Hughes, Guthrie Birkhead, D. Peter Drotman.

For me (D.P.D.), Dr Lederberg was a great friend and early champion of EID, supporting the journal from its first issue in 1995. He helped us obtain credibility by recruiting editorial board members, supporting our application for early listing in national databases, and encouraging authors and reviewers of the nascent journal. We profiled him briefly when we published his plenary lecture at the inaugural International Conference on Emerging Infectious Diseases (Figure 3) (*2*) and compared his far-seeing work to that of Rudolf Virchow, one of the founders of modern medical science, because both wrote reports that called the attention of central governments to the manifold contributors to the web of causation of emerging diseases (*10*).

Dr Lederberg will be remembered for his scientific vision and contributions, his integrity and credibility, and his unwavering commitment to advocate for the highest quality science and evidence-based public policy. He was a firm believer in the need for scientists and public health officials to communicate clearly and concisely with policy makers and the public on scientific and public health issues. His influence and impact reached broadly, across areas of expertise and around the world. He will be greatly missed.
